# Metabolic progression to clinical phenotype in classic Fabry disease

**DOI:** 10.1186/s13052-016-0320-1

**Published:** 2017-01-03

**Authors:** Marco Spada, David Kasper, Veronica Pagliardini, Elisa Biamino, Silvana Giachero, Francesco Porta

**Affiliations:** 1 Department of Pediatrics, University of Torino, Piazza Polonia 94, 10126 Torino, Italy; 2 Archimed Life Science GmbH, Vienna, Austria

**Keywords:** Fabry disease, Globotriaosylsphingosine, Lysosomal storage disorders

## Abstract

**Background:**

Fabry disease is an X-linked lysosomal storage disorder due to α-galactosidase A (α-Gal A) deficiency. Clinical onset of Fabry disease is preceded by significant storage of globotriaosylceramide (Gb3) and related glycosphingolipids, but the extent of the metabolic progression before symptoms is unknown. Using a newly recognized effector and marker of Fabry disease, globotriaosylsphingosine (LysoGb3), we aimed to provide a metabolic picture of classic Fabry disease from the neonatal period to childhood.

**Methods:**

LysoGb3 was assessed at different times in two brothers with classic Fabry disease (genotype c. 370–2 A > G). The firstborn was diagnosed after clinical onset at 11 years of age, whereas the second-born was diagnosed in the neonatal period. LysoGb3 was measured in dried blood spots by high-sensitive electrospray ionization liquid chromatography tandem mass spectrometry.

**Results:**

Blood LysoGb3 concentrations were consistent with patients’ age and clinical picture, with lower levels in the asymptomatic neonate (19.1 ng/ml) and higher levels in the symptomatic child (94.3 ng/ml). In the second-born, LysoGb3 doubled during the first 5 months of life (37.4 ng/ml), reaching ~40% concentration observed in the symptomatic period. The neonatal LysoGb3 concentration in classic Fabry disease exceeded that observed in normal subjects by over 15 times.

**Conclusions:**

A substantial increase of LysoGb3 was documented during the first months of life in classic Fabry disease, suggesting an early plateau during the pre-symptomatic period. Such a progressive metabolic trend during the pre-symptomatic period implies the potential definition of a metabolic threshold useful for a preventive therapeutic approach of classic Fabry disease. Additionally, the consistent increase of LysoGb3 in the neonatal period in classic Fabry disease suggests LysoGb3 as a useful marker for improving the specificity of newborn screening for Fabry disease.

## Background

Fabry disease (OMIM 301500) is an X-linked lysosomal storage disorder due to α-galactosidase A (α-Gal A) deficiency. The enzymatic defect leads to progressive accumulation of globotriaosylceramide (Gb3) and related glycosphingolipids in the vascular endothelium, particularly in kidney, brain, and heart. Affected males with complete or near-complete α-Gal A deficiency exhibit the classic clinical phenotype of Fabry disease with onset of angiokeratomas, acroparesthesias, hypohidrosis, and corneal opacities in childhood, followed by renal failure, cardiac and cerebrovascular disease, and premature death [1, 2]. A wide spectrum of later-onset variants have been described in patients with residual α-Gal A activity, including the “renal variant” and the “cardiac variant”. Since 2001, an effective enzyme replacement therapy (ERT) is available [3].

Recently, globotriaosylsphingosine (LysoGb3), a deacylated form of Gb3, was identified as a new pathogenetic effector and hallmark of Fabry disease, representing a promising non-invasive marker for monitoring the disease [4]. Differently from plasma Gb3, plasma LysoGb3 was shown to be dramatically increased in both males with classic Fabry disease and symptomatic females heterozygous for mutations in the α-galactosidase A gene [5]. Since the clinical phenotype of Fabry disease is invariably preceded by earlier progressive lysosomal storage of glycosphingolipids [6], the analysis of peripheral LysoGb3 may allow investigation of patients’ metabolic phenotype even in the pre-symptomatic period.

Here we report LysoGb3 analysis in two brothers with classic Fabry disease with 14 years age difference, giving a picture of metabolic phenotype and natural progression of Fabry disease from the neonatal period to childhood.

## Methods

**Fig. 1:**
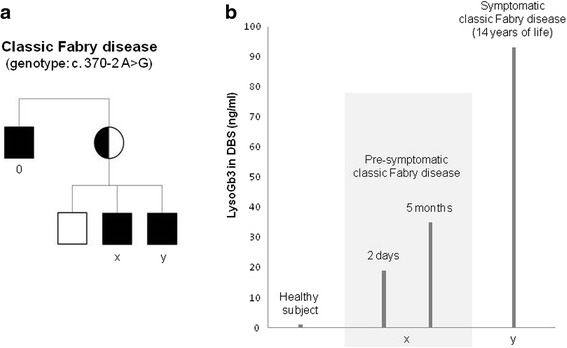
**a**, Familial pedigree of two brothers (“x” and “y”) with classic Fabry disease (details in text). Patient “0” was diagnosed with end-stage renal disease due to classic Fabry disease at 35 years of age. **b**, Globotriaosylsphingosine (LysoGb3) assessed on dried blood spot (DBS) in two brothers (“x” and “y”) at different metabolic and clinical stages of classic Fabry disease

The genealogy of the two brothers with classic Fabry disease is depicted in Fig. 1 (panel a). The clinical course of the first-born brother was uneventful until 11 years of age, when persistent acroparesthesias and burning pain were reported. A definite diagnosis of classic Fabry disease was made on the basis of biochemical and molecular data (α-Gal A activity = 0.2 nmol/h/ml, normal value >2 nmol/h/ml; genotype c. 370–2 A > G). A basal LysoGb3 analysis on dried blood spot was obtained just before starting ERT. Based on familiar anamnesis, the second-born brother was diagnosed with classic Fabry disease in the neonatal period (α-Gal A activity = 0.7 nmol/h/ml; normal value >2 nmol/h/ml; genotype c. 370–2 A > G), undergoing LysoGb3 analysis at 2 days of life. A further LysoGb3 measurement was performed at 5 months.

LysoGb3 was measured in dried blood spot samples by high-sensitive electrospray ionization liquid chromatography tandem mass spectrometry (ESI LC-MS/MS). A 7-point serum calibrator for lysoGb3 quantification (covering the analytic range from 0–120 ng/mL; lower limit of quantification: 1.5 ng/mL) and three calibrator levels (3, 30 and 100 ng/mL) for quality control were used (ARCHIMED Life Science GmbH, Vienna, Austria; www.archimedlife.com).

## Results

Blood LysoGb3 concentrations were consistent with patients’ age and clinical picture, with lower levels in the asymptomatic neonate and higher levels in the symptomatic child. LysoGb3 in the second-born doubled during the first 5 months of life, reaching ~40% concentration observed in the symptomatic period (Fig. 1, panel b). The comparison of LysoGb3 concentrations in the two brothers with classic Fabry disease revealed its 5-fold increase from the neonatal period to childhood (Fig. 1, panel b). The neonatal LysoGb3 concentration in classic Fabry disease, moreover, exceeds that observed in normal subjects by over 15 times (Fig. 1, panel b).

## Discussion

In 2006, we were the first to demonstrate a high incidence of later-onset Fabry disease as opposed to classic Fabry disease [7], subsequently confirmed in other studies [8, 9]. A variable symptom-free interval characterizes all forms of Fabry disease, and clinical phenotype is preceded and sustained by progressive glycosphingolipids storage [3]. In recent years, little was known about pre-symptomatic Fabry disease, especially since invasive procedures were invariably required for any pathological assessment. For instance, serial renal biopsies showed that Gb3 storage even precedes microalbuminuria in patients with Fabry disease [10]. Recently, podocyturia was identified as an early useful marker of renal damage in Fabry disease [11, 12].

LysoGb3 is a new, easily measurable marker and pathogenic effector of Fabry disease [4]. Smid and colleagues recently described the superior diagnostic utility of lysoGb3 compared to Gb3 to discern non-classical, uncertain or patients having no Fabry disease [13].

LysoGb3 can be measured in plasma or in dried blood spots [14], representing a potential new avenue to the comprehension of metabolic progression of Fabry disease from the pre-symptomatic to the symptomatic period. This potential of LysoGb3, indeed, may be useful for monitoring patients detected through newborn- or selective-screening for Fabry disease, improving the sensitivity of the clinical approach to prevent irreversible organ damage.

In this study, the comparison of LysoGb3 in two brothers with complete α-Gal A deficiency describes the extent of glycosphingolipids storage during childhood in classic Fabry disease and its relationship with clinical onset. The early and consistent increase of LysoGb3 observed in the neonatal period in classic Fabry disease is consistent with its fetal storage [6], making LysoGb3 a potential useful tool for improving the specificity of newborn screening for Fabry disease. Moreover, a substantial increase of LysoGb3 was documented during infancy in classic Fabry disease, suggesting an early plateau during the pre-symptomatic period. The observation of such a progressive metabolic trend in pre-symptomatic and early-symptomatic patients with classic Fabry disease implies the potential definition of a metabolic threshold (i.e. a LysoGb3 cut-off) useful for addressing an early preventive therapeutic approach to Fabry disease.

## Conclusions

These observations suggest a new nosological classification of Fabry disease, based on the metabolic phenotype instead of the clinical phenotype. Early screening for Fabry disease and longitudinal pre-symptomatic non-invasive biochemical monitoring are essential to this definition. Anticipating clinical attention on patients’ metabolic phenotype may represent a new frontier for the optimization of medical management of Fabry disease.

## Abbreviations

(ERT): Enzyme replacement therapy
(Gb3): Globotriaosylceramide
(LysoGb3): Globotriaosylsphingosine
(α-Gal A): α-galactosidase A
